# The Gut Microbiota of Pheasant Lineages Reflects Their Host Genetic Variation

**DOI:** 10.3389/fgene.2020.00859

**Published:** 2020-08-04

**Authors:** Jinmei Ding, Ting Jiang, Hao Zhou, Lingyu Yang, Chuan He, Ke Xu, Fisayo T. Akinyemi, Chengxiao Han, Huaixi Luo, Chao Qin, He Meng

**Affiliations:** Shanghai Key Laboratory of Veterinary Biotechnology, Department of Animal Science, School of Agriculture and Biology, Shanghai Jiao Tong University, Shanghai, China

**Keywords:** gut microbiota, pheasant, genetic variation, host phylogeny, microbial dendrogram, composition

## Abstract

The host gut colonized enormous microbial community, which can be influenced by diet, diseases, behavior, age, gender, hereditary effects, and environmental factors. However, the relationship between gut microbiota and host genetic variation has not yet been elucidated. In this study, we chose five pheasant lineages—Ring-necked pheasant (RN), Manchurian pheasant (MX), Phasianus versicolor (PV), Shenhong pheasant (SP), and Melanistic mutant pheasant (MM)—to investigate the gut microbial composition of pheasants and its relationship with host genetic variation. Microbial classifications revealed 29 phyla and 241 genera presented in pheasants, with the dominant phylum of *Firmicutes* and the genus of *Lactobacillus*. Statistical analyses suggest that the relative abundance of 75 genera was significantly different among the five lineages. The most abundant genus carried by the RN and MM was *Streptococcus*, which was significantly lower in PV (*p* = 0.024). Conversely, *Lactobacillus* was the major genera in PV and MX. Moreover, the RN had the greatest microbial abundance, with a remarkably different microbial community than PV. The gut microbial diversity of PV was the lowest and diverged significantly from the RN and MX. Interestingly, the clustering of the MM and SP in the microbial dendrogram corresponded to their cluster in the host phylogeny. The host phylogenetic split of the RN, MX, and PV echoed their microbial distance. In conclusion, the congruence of host phylogeny and their gut microbial dendrograms implies that gut microbiota of pheasant lineages could reflect their host genetic variation.

## Introduction

Animals are colonized by complex gut microbiota that play a crucial role in the physiological metabolism, growth, development, and evolution of the host (Turnbaugh and Gordon, 2009; Turnbaugh et al., 2009), and the structure of microbial community can be shaped by environmental factors and host genetic variations (Ley et al., 2006). The interaction between host organisms and gut microbiota has been studied and reported (Nicholson et al., 2012). Gut microbiome compositions show significant similarities in genetically unrelated individuals when they share a relatively common environment (Rothschild et al., 2018). Furthermore, imbalances and perturbations of gut microbiota that are influenced by the environment typically cause a number of diseases in humans and animals, including obesity, inflammatory bowel disease, and autism (Turnbaugh et al., 2006; Marchesi et al., 2007; Hsiao et al., 2013). Germ-free animals, who lack any bacterial colonization, display defects in the ability to fight infections induced by pathogenic bacteria and viruses (Shanmugam et al., 2005; Slack et al., 2009). Likewise, host evolutionary history determines the prevalence of specific microbial taxa in mammals (Youngblut et al., 2019). The relative abundance of specific gut microbial members can be shaped by the host’s genotype (Goodrich et al., 2016a). Research has shown that genome-wide markers have been associated with the beta diversity of the microbiome in humans (Ma et al., 2014; Blekhman et al., 2015), and variations in the copy number of the human salivary amylase gene AMY1 influences the diversity and function of the human oral and gut microbiome (Poole et al., 2019). Moreover, mouse knockout experiments identified genes involved in immunity, metabolism, and behavior that affect gut microbiota (Spor et al., 2011). And when compared to dizygotic twins, monozygotic twins tend to have more similar microbial communities (Goodrich et al., 2016b). Additionally, a study of hydra indicated that the microbiota of polyps raised in the laboratory for more than 30 years maintained similar characteristics with wild polyps, even over the long time (Fraune and Bosch, 2007). These significant congruence indicate phylosymbiosis, which is when the phylogeny of host species parallels the ecological relatedness of corresponding microbial communities (Brooks et al., 2016; Ross et al., 2018). Considering that the host genetic background plays an important role in gut microbial colony structure, gut microbiota perhaps reflects their host genetic variation underlying observations of phylosymbiosis. Therefore, we aimed to investigate this unclear mechanism by analyzing the gut microbiota composition and function in different pheasant lineages.

Pheasant (*Phasianus colchicus*, NCBI Taxon ID: 9054) is an important member of birds in the genus *Phasianus*, within the order of Galliformes and family of Phasianidae, and different from chickens, which are a subspecies of the red junglefowl, belonging to the genus *Gallus*. Pheasants have 30 subspecies in the world, and about two-thirds of these subspecies are widespread throughout China. Although native to Asia, pheasants were widely introduced elsewhere in the first century BC as a game bird because of their distinguished colorful feathers, good motion performance, fast growth ability, and high levels of disease resistance (Shen et al., 2009; Gu et al., 2016). The hardy pheasants adapt readily to the wild and are prized by sportsmen for their excellent flying ability and because they are tolerant of extreme cold and heat conditions (Madden et al., 2018). The pheasants are increasingly used for adaptive breeding and research because of their high nutritional value, favorable egg quality, meat production performance, and high economic efficiency (Aldous and Alexander, 2008; Naish, 2011).

For this study, we chose five pheasant lineages, including the ring-neck pheasant (RN), manchurian pheasant (MX), phasianus versicolor (PV), Shenhong pheasant (SP), and melanistic mutant pheasant (MM), which were all raised in the Shanghai Xinhao rare poultry breeding company in China. All lineages of them were maintained at the same location and reared on the same diets from 2012. In the past, all of these lineages experienced different selection pressures through either natural or artificial selection. The RN, MX, and PV were imported to China from MacFarlane Pheasants, Inc. of the United States in 2012. SP and MM were long-term domestic pheasant lines on the Chinese farm. The RN is the most popular of the pheasant lines and is primarily used for stocking and hunting. They are also often used by clubs and growers for meat production. The MM is a pure line, distinguished by its large, beautiful pheasant feature and its iridescent, greenish-black plumage; it also displays a remarkable ability to survive and reproduce in the wild. Compared with the RN and the MX, the domestic SP has significant advantages in terms of egg weight, yolk size, and yolk color grade. Considering the varying population characteristics and different genetic backgrounds, these five pheasant lines were selected as the ideal experimental model in order to investigate the relationship between gut microbiota and host genetic variation.

## Results

### Characterization of Pheasant Gut Microbiota

**FIGURE 1:**
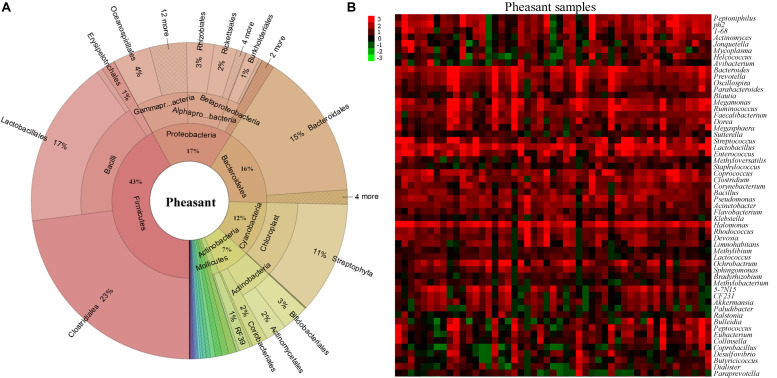
Aggregate gut microbiota composition. **(A)** The distribution of the pheasant gut microbiota. **(B)** Heatmap of the pheasant gut microbiota at the genus level. Colors reflect relative abundance from low (green) to high (red).

To understand how the gut microbiome are shaped in different pheasants, we collected 49 pheasant fecal samples. After high-throughput sequencing of their DNA, we obtained a total of 30,313 operational taxonomic units (OTUs), of which 4,340 were quality filtered and classified as different microbes. Subsequently, the microbial classifications revealed that 29 phyla were present in the pheasants. *Firmicutes* was the predominant phylum (43%), followed by *Proteobacteria* (17%), *Bacteroidetes* (16%), and *Cyanobacteria* (12%) (Figure 1A). Among them, the ratio of *Firmicutes* to *Bacteroidetes* was about 3:1 (Supplementary Figure S1A). According to the quality-filtered OTUs, 80% were classified into 169 families. The most abundant families found in the pheasants were *Lactobacillaceae* (9%), *Tissierellaceae* (8%), *Streptococcaceae* (7%), *Bacteroidaceae* (6%), and *Ruminococcaceae* (5%), with all other families found to be present at average levels of <5% (Supplementary Figure S1B). At the genus level, 241 genera were detected. In addition to *Lactobacillus, Halomonas*, and *Bacteroides*, we found that *Coprococcus, Enterococcus*, and *Streptococcus* were also dominant genera (Figure 1B). Some of the short-chain fatty acid producing genera, such as *Bacteroides, Faecalibacterium, Blautia, Coprococcus, Clostridium*, and *Ruminococcus*, are highly abundant in pheasants (Figure 1B).

### Gut Microbial Diversity and Composition in Different Pheasant Lineages

**FIGURE 2:**
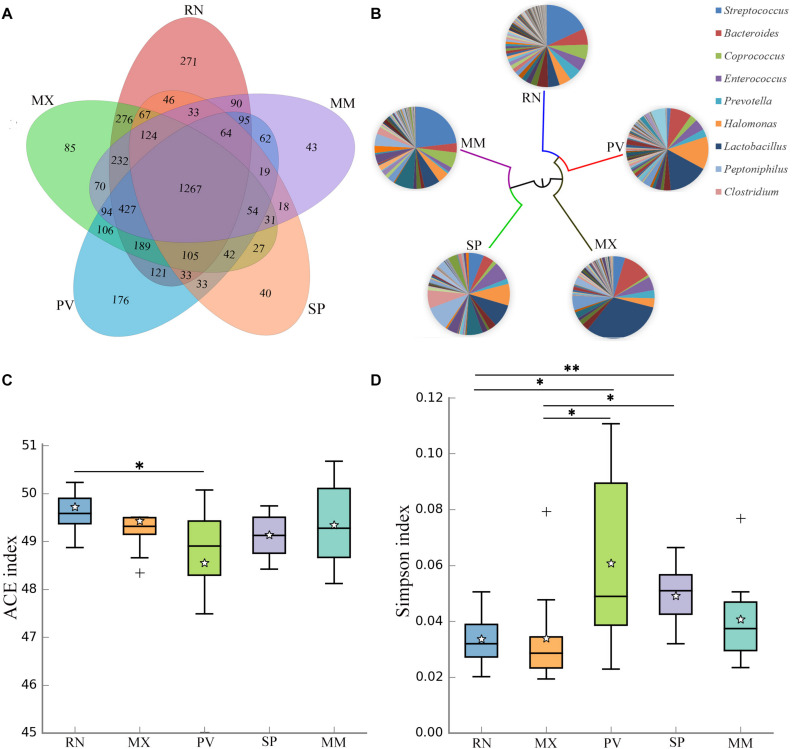
Gut microbial communities in different pheasant lineages [ring-necked pheasant (RN), manchurian pheasant (MX), phasianus versicolor (PV), Shenhong pheasant (SP), and melanistic mutant pheasant (MM)]. **(A)** The Venn diagram shows the OTUs shared within different lines. **(B)** Aggregate microbiota composition and dendrogram at different lineages. Only major taxonomic groups are shown in pie chart. **(C)** Alpha diversity of ACE index in five pheasant lineages. **(D)** Alpha diversity of Simpson index in five pheasant lineages. ^∗^*p* < 0.05 and ^∗∗^*p* < 0.01.

The genetic lines of the RN, MX, PV, SP, and MM were included in this study, which found that 29% of the OTUs were shared by these five pheasant lines (Figure 2A). Microbial dendrogram of weighted UniFrac distances (Goodrich et al., 2016b) constructed from the gut microbiota revealed that the gut microbial evolutionary relationship of the RN, MX, and PV were closely related (Figure 2B). The microbial distance of the MM was further from the other pheasants. We also compared the gut microbial diversity and richness between these five lineages. The RN had the greatest microbial abundance, which was remarkably different from PV (Figure 2C). The gut microbial diversity of PV was the lowest and significantly divergent from the RN and MX (Figure 2D).

A total of 29 phyla, 241 genera, and 95 species were identified in these five lines, and five major phyla dominated the pheasant gut microbial community (Supplementary Figure S2). There were 11 phyla that differed among the RN, MX, PV, SP, and MM (Table 1). One of the abundant phyla in all lines was *Firmicutes*, which had a significant difference between PV and MM (*p* = 0.031). Compared with the MX, *Proteobacteria* were more prevalent in PV (*p* = 0.027). *Actinobacteria, Synergistetes*, and *Thermotogae* were different for MX and MM. *Crenarchaeota* and *Chloroflexi* were more enriched in RN than in PV (*p* = 0.042) and SP (*p* = 0.012), respectively (Table 1). At the genus level, the pie chart showed an obvious distinction of gut microbiota in these five lines (Figure 2B). Among the 241 genera, 75 were conspicuously different in the different lines (Supplementary Table S1). The most abundant genus carried by the RN and MM was *Streptococcus*, which showed a significantly low percentage in PV (*p* = 0.024) (Supplementary Figure S3 and Supplementary Table S1). Conversely, *Lactobacillus, Halomonas, Bacteroides*, and *Veillonella* were the major genera in PV (Figure 2B). When compared to the RN and MX lines, *Halomonas* showed greater richness in PV (*p* < 0.05). *Lactobacillus* was enriched in the MX (32%), while it accounted for only 5% in the RN (Supplementary Figure S3). For SP, the dominant microbial community was distributed evenly (Figure 2B). Moreover, we also analyzed the gut microbiota at the species level. According to the filtered OTUs, only 6% of species were identified in current technology. While we observed 95 species, 39 of them were remarkably different among the lines (Supplementary Figure S4A and Supplementary Table S2). The species *alactolyticus*, which belongs to the genus *Streptococcus*, was deficient in PV, where it was significantly lower than in the RN (*p* < 0.05), SP (*p* < 0.01), and MM (*p* < 0.05) (Supplementary Figure S4B). Conversely, *Veillonella dispar* and *Haemophilus parainfluenzae* were enriched only in PV. Similarly, *Lactobacillus agilis* and *Bacillus cereus* were only sufficient in SP (Supplementary Figure S4).

**TABLE 1:** The significantly different (*p* < 0.05) gut microbial relative abundance at the phylum level among five pheasant lineages.

**Phylum**	**Group 1 (mean ± SE)**	**Group 2 (mean ± SE)**	***p-*value (group 1 vs. group 2)**
*Firmicutes*	PV (13.8 ± 0.55)	MM (15.1 ± 0.21)	0.031
*Proteobacteria*	MX (12.3 ± 0.68)	PV (14 ± 0.28)	0.027
*Actinobacteria*	MX (10.62 ± 0.48)	MM (12.51 ± 0.38)	0.018
*Crenarchaeota*	RN (8.1 ± 0.99)	PV (5.3 ± 1.17)	0.042
*Synergistetes*	MX (4.39 ± 1)	MM (7.8 ± 0.96)	0.018
*Chloroflexi*	RN (7 ± 0.83)	SP (3.71 ± 0.82)	0.012
*TM7*	PV (2.56 ± 0.89)	MM (6.07 ± 0.52)	0.002
	MX (3.81 ± 0.59)	MM (6.07 ± 0.52)	0.045
*WS3*	RN (3.28 ± 1.07)	SP (1.02 ± 0.57)	0.05
*SAR406*	MX (2.97 ± 0.63)	SP (0 ± 0)	0.008
	PV (2.96 ± 1.02)	SP (0 ± 0)	0.008
	SP (0 ± 0)	MM (2.55 ± 0.81)	0.021
	RN (0.88 ± 0.57)	MX (2.97 ± 0.63)	0.05
*Thermotogae*	MX (1.77 ± 0.58)	PV (0 ± 0)	0.004
	MX (1.77 ± 0.58)	SP (0 ± 0)	0.005
	RN (1.71 ± 0.56)	PV (0 ± 0)	0.005
	RN (1.71 ± 0.56)	SP (0 ± 0)	0.006
	MX (1.77 ± 0.58)	MM (0.3 ± 0.28)	0.014
	RN (1.71 ± 0.56)	MM (0.3 ± 0.28)	0.018
*CD12*	RN (1.4 ± 0.42)	PV (0.1 ± 0.09)	0.007
	RN (1.4 ± 0.42)	SP (0.11 ± 0.1)	0.009
	RN (1.4 ± 0.42)	MX (0.36 ± 0.22)	0.029

### Predicted Functions of Gut Microbiota Varied in the Different Pheasant Lines

**FIGURE 3:**
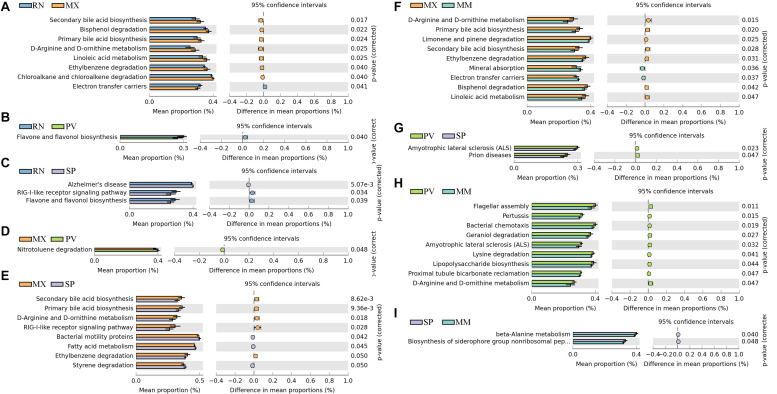
Significant differences in microbial metabolic pathways at ring-necked pheasant (RN), manchurian pheasant (MX), phasianus versicolor (PV), Shenhong pheasant (SP), and melanistic mutant pheasant (MM). **(A)** RN versus MX. **(B)** RN versus PV. **(C)** RN versus SP. **(D)** MX versus PV. **(E)** MX versus SP. **(F)** MX versus MM. **(G)** PV versus SP. **(H)** PV versus MM. **(I)** SP versus MM.

We identified differences in functional pathways between these five lines using PICRUSt prediction (Figure 3). By comparing the pairwise overlap, at the three-level functional pathway, we discovered that the RN harbored microbiota with increased electron transfer carriers as compared to MX, and with more enriched RIG-I-like receptor signaling pathway than SP (Figures 3A–C). Flavone and flavonol biosynthesis, which benefits organisms due to their diverse biological and pharmacological activities in hepatoprotection, anti-oxidation, anti-mutagenesis, anti-inflammation, anti-viral, and against coronary heart diseases, was also more highly enriched in RN than PV and SP (Spor et al., 2011). In MX, there was a greater proportion than RN, SP, or MM of pathways involved in microbial functions relating to secondary bile acid biosynthesis, primary bile acid biosynthesis, linoleic acid metabolism, ethylbenzene degradation, chloroalkane and chloroalkane degradation, bisphenol degradation, d-arginine and d-ornithine metabolism, and RIG-I-like receptor signaling (Figures 3A,E,F). In PV, the pathways of amyotrophic lateral sclerosis and prion diseases were significantly greater than in SP. Notably, the microbiota in PV had a greater abundance of functional capacities involved in cell motility, such as flagellar assembly and bacterial chemotaxis, than in MM. Likewise, nitrotoluene degradation, lysine degradation, geraniol degradation, and lipopolysaccharide biosynthesis were also enriched in PV (Figures 3D,G,H). In comparison to MM, we observed more microbes in SP participating in the functions of beta-alanine metabolism and biosynthesis of siderophore group non-ribosomal peptides (Figure 3I).

### Phylosymbiosis Occurred in Different Pheasant Lineages

**FIGURE 4:**
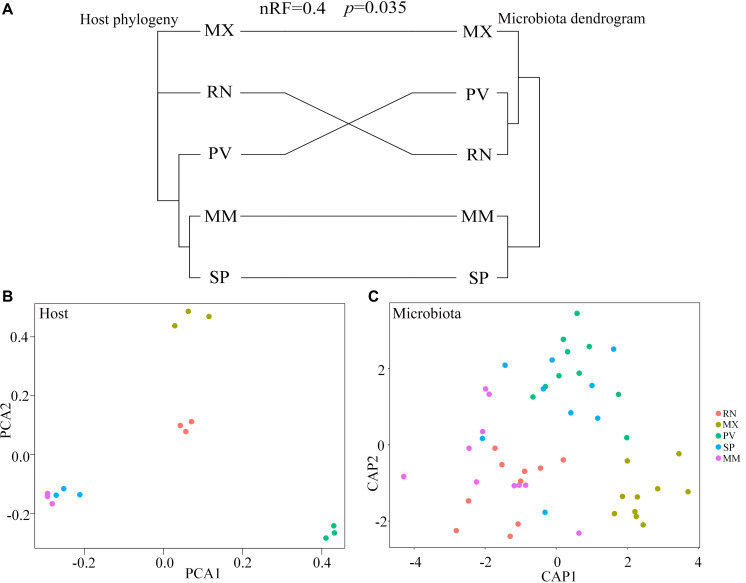
Phylosymbiosis occurred in ring-necked pheasant (RN), manchurian pheasant (MX), phasianus versicolor (PV), Shenhong pheasant (SP), and melanistic mutant pheasant (MM). **(A)** Comparing host phylogeny to dendrogram of corresponding microbial communities for five pheasant lines. Host phylogeny constructed using filtered SNPs of pheasant whole genome. Microbial dendrogram were constructed using beta diversity. Congruence was measured using normalized Robinson–Foulds (nRF) scores. **(B)** Principal component analysis (PCA) based on the host genome. **(C)** Canonical analysis of principal coordinates (CAP) based on the weighted UniFrac metrics of gut microbiota to reveal a separation among lineages.

Comparing host phylogeny to the dendrogram of corresponding microbial communities for each line of pheasant indicated that relationships among gut microbiota were similar to their corresponding pheasant evolutionary relationships (Figure 4A). Phylosymbiosis was exhibited by the closely related clades of pheasants having an association with microbiota that had similar thresholds and distance measures. A Robinson–Foulds score of 0 indicates that the tree is identical, whereas a score of 1 indicates there is no congruence between the two trees. In this study, the Robinson–Foulds score of 0.4 indicated significant congruence between the pheasant phylogeny and microbial dendrogram established with weighted UniFrac metrics. The similar clustering of MM and SP phylogeny and microbial dendrogram can be attributed to the same long-term husbandry conditions (Figure 4A). Principal component analysis (PCA) also showed that MM and SP were closely related, which further demonstrated that host phylogeny can be influenced by their similar origins (Figure 4B). This result also corresponds to the gut microbial background of MM and SP. Canonical analysis of principal coordinates based on the weighted UniFrac metrics also revealed an obvious separation among lines (Figure 4C). The largest discrepancy was observed for RN, which grouped with PV but not MX (Figure 4A). In addition, there was a split of RN, MX, and PV in phylogeny that was echoed by the microbial distance of these three lines (Figures 4B,C).

## Discussion

Each host species and all of its symbiotic microorganisms were collectively described as holobionts (Mindell, 1992; Margulis, 1993). The sum of the genetic information of a holobiont was defined as a hologenome (Zilber-Rosenberg and Rosenberg, 2008). The microbial symbionts and the host interact in ways that affect the physiology, health, and fitness of the holobiont within its environment. The totality of their interactions characterizes the holobiont as a unique biological entity and, therefore, also as a level of selection in evolution (Rosenberg et al., 2007). Genetic variation in the hologenome can be brought about by changes in either the host genome or the microbiome. In previous studies, significant genetic correlations of microorganisms have been observed in the body weight and abdominal fat pad weight selection lines of chickens (Zhao et al., 2013; Ding et al., 2016). Further analysis showed that such genetic correlations can be altered by genetic variation of the host (Meng et al., 2014). Under environmental change and selection stress, the microbiome can adjust more rapidly, allowing for more enhancement of the holobiont evolution than could be accomplished by the host organism alone. In the study of long-term divergent antibody selection, gut microbiota have been shown to respond rapidly to changes in host genomes and undergo adaptive changes, indicating that the variation may have an important impact on the differences of host traits (Yang et al., 2017). Therefore, gut microbiota may be an indicator and reflection of host adaptation and evolution. In our study, the RN, MX, and PV were imported from the same farm at the same time, and SP and MM were long-term domestic pheasant lines, but all of these lines had been maintained in the same husbandry conditions for several years. The lines of MX, PV, and RN clustered together in the gut microbial dendrogram may suggest that the composition of gut microbiota can be influenced by the environment. In contrast, there is no significant phylosymbiosis between RN phylogeny and its gut microbial dendrogram, and it is likely that the microbial dendrogram were affected by the circumstances of intensive livestock farming, where the pheasants undergo frequent feeding and spend the majority of time indoors. It may also be possible that the exceptional physical function for RN production, such as for hunting and meat production, is more suited to life in the wild, and the environmental changes are more likely to affect microbial composition in the short term but not genetics. Interestingly, the significant phylosymbiosis between phylogeny and gut microbial dendrogram in MM and SP indicates that the gut microbiota will coevolve with the host. In conclusion, the similarity of host phylogeny and dendrogram of its gut microbiota indicate that phylosymbiotic relationships exist in holobionts and their hologenome evolutionary relationships. This phylosymbiosis implies that the host genetic variation could be reflected by its gut microbiota.

Alpha diversity analysis revealed high levels of gut microbial community richness and diversity in the pheasants (Supplementary Figure S5). Compared with the 17 phyla found in chickens and nine phyla found in pigs in prior studies (Liu et al., 2015; Ding et al., 2017), the microbial classification in the current study revealed that 29 phyla were present in pheasants (Figure 1). Like humans, chickens, and pigs, *Firmicutes* was dominant in the gut microbiota of the pheasants as well (Backhed et al., 2015; Zhao et al., 2015; Wen et al., 2019). However, in humans and other animals, *Cyanobacteria* do not exhibit the high abundance found in the pheasants (12%). *Cyanobacteria* is a phylum of bacteria that use the energy of sunlight to drive photosynthesis, which is the synthesis of organic compounds from carbon dioxide, and *Cyanobacteria* are the only photosynthetic prokaryotes able to produce oxygen. In our study, photosynthesis-antenna proteins, which fall into the category of energy metabolism, appeared at high levels of richness in the pheasants (Supplementary Figure S6). Energy homeostasis plays a well-understood pivotal role in the survival of organisms living in a diverse environment. Photosynthesis-antenna proteins usually exist in phycobilisomes or light-harvesting chlorophyll protein complexes in green plants, and act as peripheral antenna systems enabling more efficient absorption of light energy. Thus, the abundance of photosynthesis-antenna proteins in pheasants may be due to the *Cyanobacteria* (Supplementary Figure 1A). Furthermore, the number of genera observed in the pheasants was twice that found in chickens (Supplementary Figure S5). Host genetics may be the cause of the differences in genera found in the pheasants and in chickens, such as the fivefold difference for *Streptococcus*, eightfold for *Aeriscardovia*, and 16-fold for *Porphyromonas* (Supplementary Table S3). In addition, compared with chickens, there were significantly lower levels in the pheasants of some beneficial bacteria, such as *Lactobacillus, Bifidobacterium*, and butyrate-producing bacteria *Oscillospira* (Supplementary Table S3). This may be due to more strong ability of disease resistance, environmental adaptability, and diverse dietary structure in pheasants than chickens.

## Materials and Methods

### Animals and Sample Collection

Five pheasant lines were used in the study, including RN (A, *n* = 10), MX (B, *n* = 10), PV (C, *n* = 10), SP (D, *n* = 9), and MM (E, *n* = 10) from the Shanghai Xinhao rare poultry breeding company. Throughout all lineages, they were maintained at the same location and reared on the same diets from 2012. Fecal sample (*n* = 49) series were collected from all healthy pheasant individuals at 56 weeks of age with similar body weight. We also selected data on 12 fecal samples from three chicken lines (Beijing Fatty, Xianju, and Shiqiza) reported on in a published paper (Ding et al., 2017) to compare the differences between the pheasants and chickens. These samples were transported with an ice pack and immediately placed in a −80°C freezer. Animals used for this experiment were approved by Animal Welfare and Ethics Committee for the Care and Use of Laboratory Animals in Shanghai Jiao Tong University, China. In addition, we included genomic data (NCBI, project accession PRJNA380312) of another 15 samples from RN (*n* = 3), MX (*n* = 3), PV (*n* = 3), SP (*n* = 3), and MM (*n* = 3) to explore their phylogenetic relationships. The latter 15 pheasants were raised in exactly the same husbandry conditions as the previous 49 pheasants.

### Gut Microbial DNA Extraction and 16S rRNA Gene Sequencing

Gut microbial DNA was isolated from fecal samples using the TIANamp Stool DNA Kit (DP328, TIANGEN Biotech, Beijing, China) following the manufacturer’s instructions. Extracted DNA was measured using a nanodrop spectrophotometer (Thermo Fisher Scientific, Waltham, MA, United States) to assess DNA quantity and quality. The V4 hypervariable region of the 16S rRNA gene was PCR-amplified from genomic DNA using sample-specific sequence barcode fusion primers (forward 5′AYTGGGYDTAAAGNG 3′, reverse 5′TACNVGGGTATCTAATCC 3′). PCR reactions and PCR product purification were performed as previously reported in Zhao et al. (2013). Purified PCR products from the 49 samples were combined at equal concentrations and used to construct a metagenomic library using the Illumina TruSeq sample preparation kit (Illumina, San Diego, CA, United States) according to the suggested protocols of manufacturers. We sequenced the 16S rRNA genes of fecal microbes using an Illumina MiSeq sequencing platform (Illumina, San Diego, CA, United States) provided by Shanghai Personal Biotechnology Co., Ltd. (Shanghai, China). The Quantitative Insights Into Microbial Ecology (QIIME, v1.8.0^1^) pipeline was employed to process the sequencing data, as previously described (Caporaso et al., 2010). Briefly, raw sequencing reads with exact matches to the barcodes were assigned to respective samples and identified as valid sequences. The low-quality sequences were filtered through following criteria (Gill et al., 2006; Chen and Jiang, 2014):sequences that had a length of <150 bp, sequences that had average Phred scores of <20, sequences that contained ambiguous bases, and sequences that contained mononucleotide repeats of >8 bp. Paired-end reads were assembled using FLASH (Magoc and Salzberg, 2011). Then, through the QIIME software call USEARCH (v5.2.236^2^), the chimeric sequences were checked and removed. The filtered sequences with an overlap longer than 10 bp between Read 1 and Read 2 and without any mismatches were assembled according to their overlapping sequences. Reads that could not be assembled were discarded. A total of 4,009,143 sequences from the V4 region of 16S rRNA sequence of 49 samples that passed our quality filters were used (Supplementary Table S4), with an average length of 225 bp for each sequence. Trimmed sequences were uploaded to QIIME (Caporaso et al., 2010) for further analysis. The sequences are publicly available from Metagenome Rapid Annotation using Subsystem Technology (MG-RAST) (Meyer et al., 2008) under the project name “Pheasant gut microbiota (mgp89286)”^3^.

### Taxonomic Assignment and Statistical Analyses

Bacterial OTUs were derived from the trimmed sequences of the PCR amplicon for the V4 hypervariable region of the 16S rRNA gene and were compared to the GreenGene databases (Desantis et al., 2006) using the uclust and blast functions in QIIME (Edgar et al., 2011). Out of the 4,009,143 amplicons, 30,313 OTUs were annotated and classified at 97% similarity from the phylum to species levels. An OTU table was further generated to record the abundance of each OTU in each sample and the taxonomy of these OTUs. OTUs containing less than 0.001% of total sequences across all samples were discarded. To minimize the difference of sequencing depth across samples, an averaged, rounded rarefied OTU table was generated by averaging 100 evenly resampled OTU subsets under the 90% of the minimum sequencing depth for further analysis. Ultimately, 4,340 OTUs were quality filtered and classified as different microbes. The OTU abundance counts were log2 transformed and normalized by subtracting the mean of all transformed values and dividing by the standard deviation of all log-transformed values for the given sample. In the end, the abundance profiles for 49 samples exhibited a mean of 0 and a standard deviation of 1. Normalized abundance was used to generate a heatmap with Cluster 3.0 and Java TreeView (de Hoon, 2002). Alpha diversity analysis was performed with the alpha-diversity.py script to calculate the ACE (Pitta et al., 2010) and Simpson (Mahaffee and Kloepper, 1997) metrics. Venn diagrams were generated using mothur (Schloss et al., 2009). Box plots and bar charts were created with SigmaPlot (Kornbrot, 2000). ANOVA with the Tukey–Kramer test and the Benjamini–Hochberg correction were chosen for multiple-group analysis (Benjamini and Hochberg, 1995).

### Microbial Function Prediction

The microbial functional profile was predicted using PICRUSt (Langille et al., 2013). The OTUs were mapped to a gg13.5 database at 97% similarity by QIIME’s command “pick_closed_otus.” The OTU abundance was normalized automatically using 16S rRNA gene copy numbers from known bacterial genomes listed in the Integrated Microbial Genomes (IMG) database. The predicted genes and their functions were aligned to the Kyoto Encyclopedia of Genes and Genomes (KEGG) (Kanehisa et al., 2014) database, and the differences among groups were compared using the STAMP software (Parks and Beiko, 2010). All *p*-values were adjusted by the Benjamini–Hochberg false discovery rate (FDR) procedure (FDR < 0.05) (Benjamini and Hochberg, 1995).

### Phylosymbiosis Analysis

The phylosymbiosis analysis of the gut microbiota and pheasant phylogeny was adapted from a published protocol (Brooks et al., 2016). The host genomes were assembled by genomic sequencing of 15 samples from five pheasant lines using MUSCLE software v3.8.31 (Edgar, 2004) and were edited by removing gap positions and 5′/3′ end overhangs with Jalview v2.9 (Waterhouse et al., 2009). The final edited alignment was created using RaxML online Blackbox server v8.2 (Stamatakis, 2006). Host phylogenetic trees were constructed through the neighbor-joining method in MEGA software using filtered single nucleotide polymorphisms (SNPs) of the whole pheasant genome in a variant call format (VCF) file. Microbial dendrogram was constructed using the QIIME v1.9.0 jackknifed_ beta_diversity.py command. Each of the above pheasant clades had gut microbiota consensus dendrogram created at 97% OTU identity threshold using weighted UniFrac distance metrics. Congruencies between host phylogenies and gut microbiota dendrogram were quantified by calculating the normalized Robinson–Foulds metric (Robinson and Foulds, 1981). These scores were calculated with the ape R package (Paradis et al., 2004) and a custom python script created by Brooks et al. (2016). The significance of these scores was determined by constructing 200,000 randomized trees with leaf nodes identical to the gut microbiota dendrogram and comparing each to the host phylogeny to calculate the number of stochastic dendrogram with equivalent or better Robinson–Foulds metrics.

## Data Availability Statement

Our data are publicly available from Metagenome Rapid Annotation using Subsystem Technology (MG-RAST) under the accession numbers mgm4843789.3 and mgm4843790.3.

## Ethics Statement

The animal study was reviewed and approved by the guidelines of the Institute for Laboratory Animal Research (ILAR) Guide for Care and Use of Laboratory Animals, Shanghai Jiao Tong University.

## Author Contributions

JD wrote the manuscript. JD, TJ, and HM conceived and designed the experimental procedure and supervised the study. JD and TJ collected samples and extracted the DNA. JD and HZ performed the statistical analysis and data processing. LY, CH, KX, FA, CH, HL, and CQ coordinated the sample collection and supervised the study. All authors read and approved the final manuscript.

## Conflict of Interest

The authors declare that the research was conducted in the absence of any commercial or financial relationships that could be construed as a potential conflict of interest.

## Footnotes

1 http://qiime.org/

2 http://www.drive5.com/usearch/

3 http://www.mg-rast.org/
