# Supplementary material for: The Gut Microbiota of Pheasant Lineages Reflects Their Host Genetic Variation
Source: Front Genet. 2020 Aug 4;11:859. doi: 10.3389/fgene.2020.00859 (PMC7438946; doi:10.3389/fgene.2020.00859)
Supplement: Supplementary file 1 [file Data_Sheet_1.docx]

Supplementary Material

## Supplementary Figures


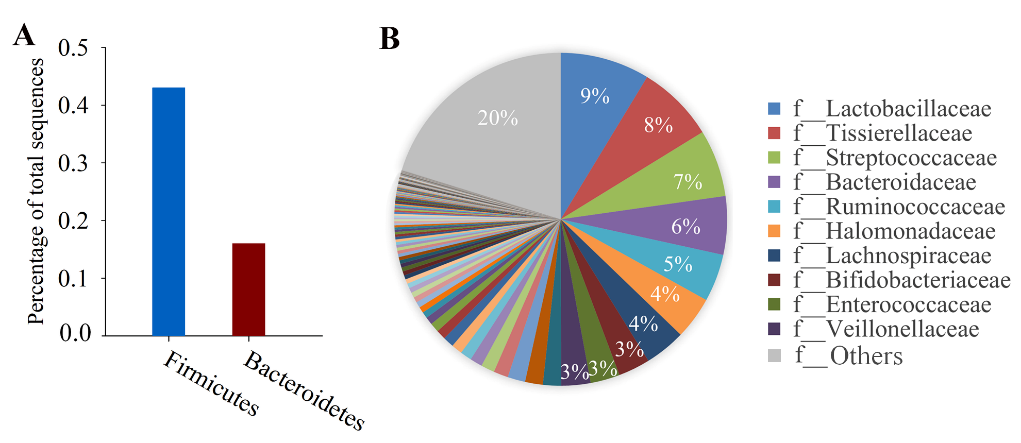


**Supplementary Figure 1. (A)** The ratio of *Firmicutes/Bacteroideres.* **(B)** The distribution of the pheasant gut microbiota at family levels.


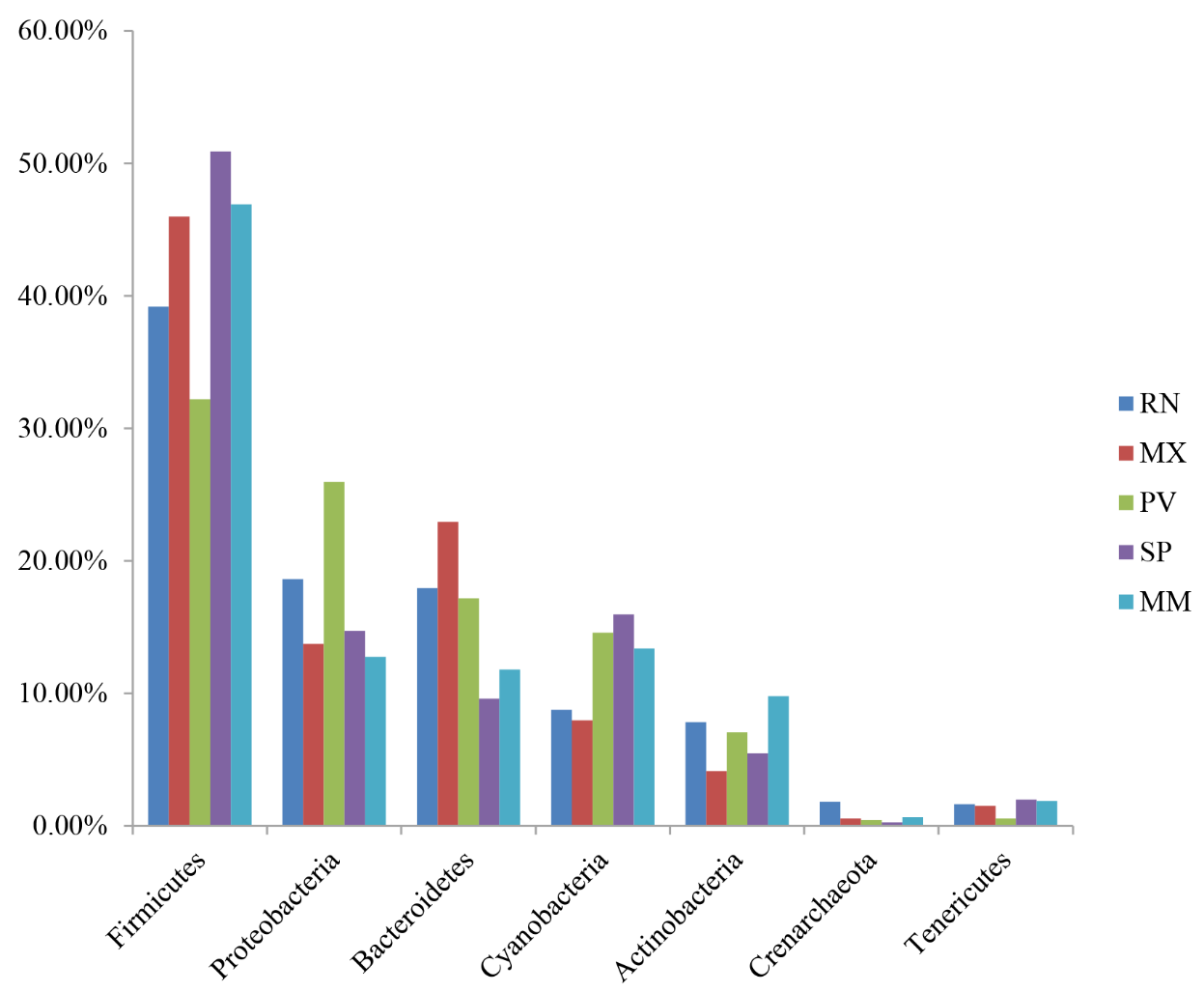


**Supplementary Figure 2** The distribution of pheasant gut microbiota at phylum levels.

**
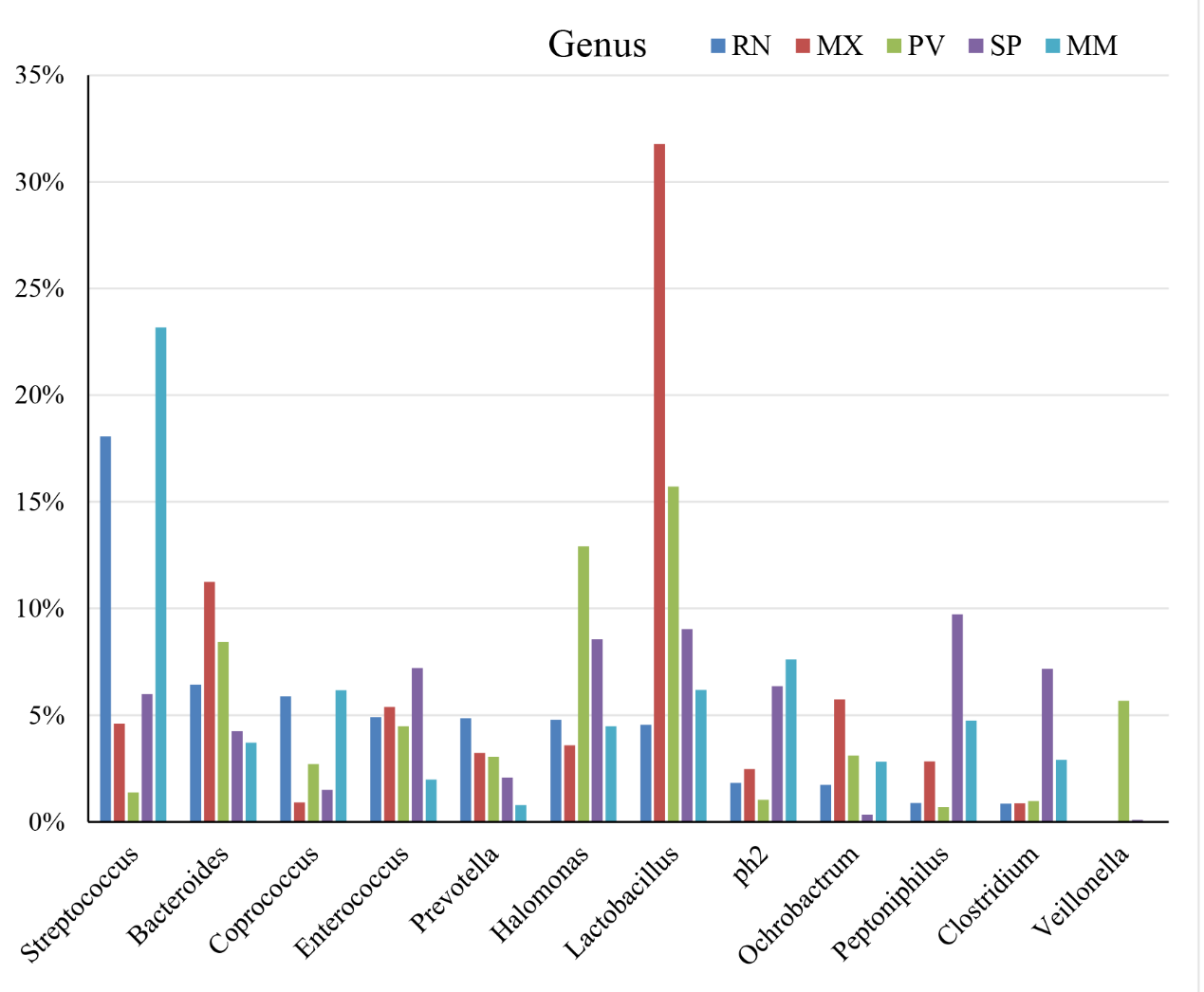
**

**Supplementary Figure 3** The distribution of pheasant gut microbiota at genus levels.

**
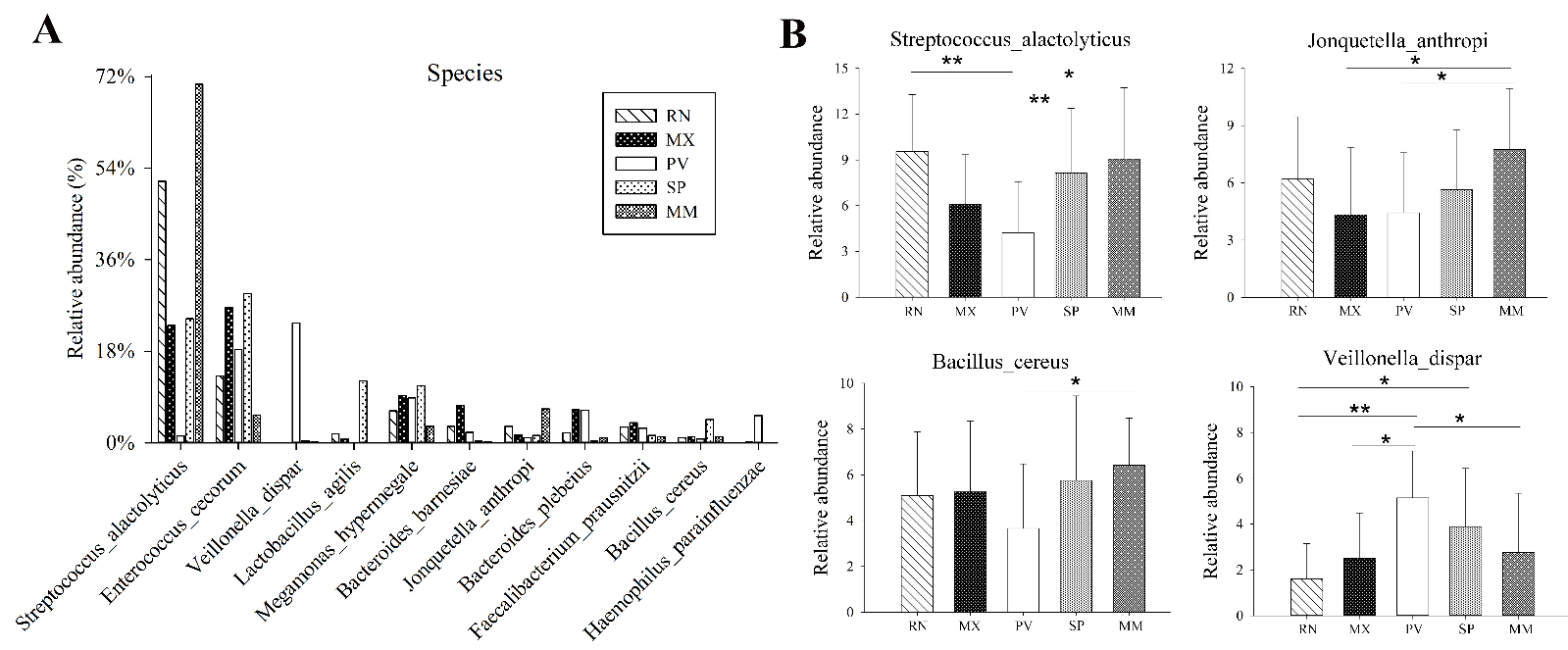
**

**Supplementary Figure 4** Gut microbial composition structure of five lines at the species level. (A) Comparisons of relative abundance at bacterial species level. (B) The remarkable different microbes within five pheasant lineages. Only major taxa are shown.­­­


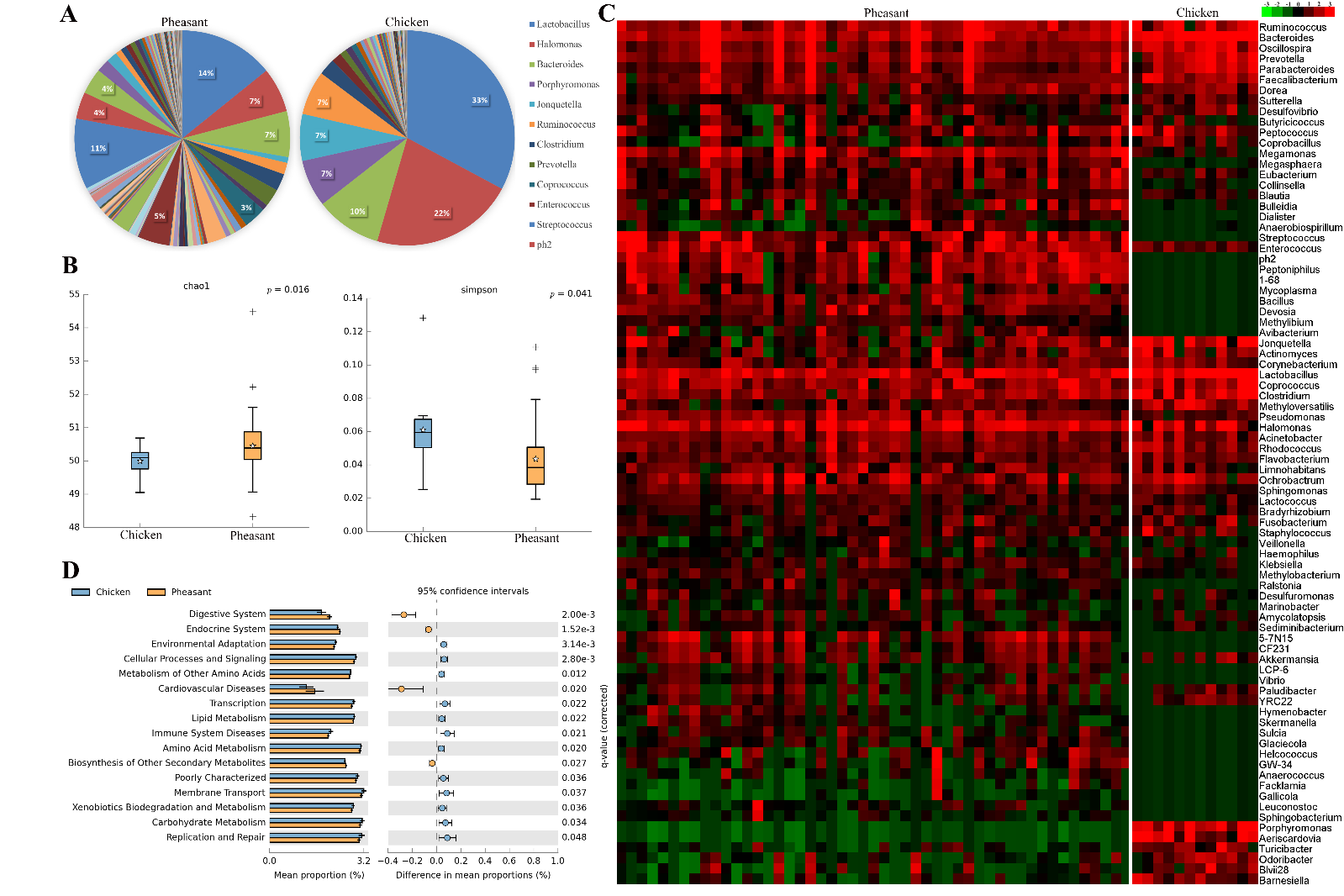


**Supplementary Figure 5** Aggregate microbiota characteristics of pheasants and chickens. **(A)** Dominant taxonomic groups of pheasants and chickens by genus. **(B)** Alpha diversity in pheasants and chickens. **(C)** Heatmap of the pheasants and chickens gut microbiota in the genus. **(D)** Significant differences in microbial metabolic pathways of the pheasants and chickens.


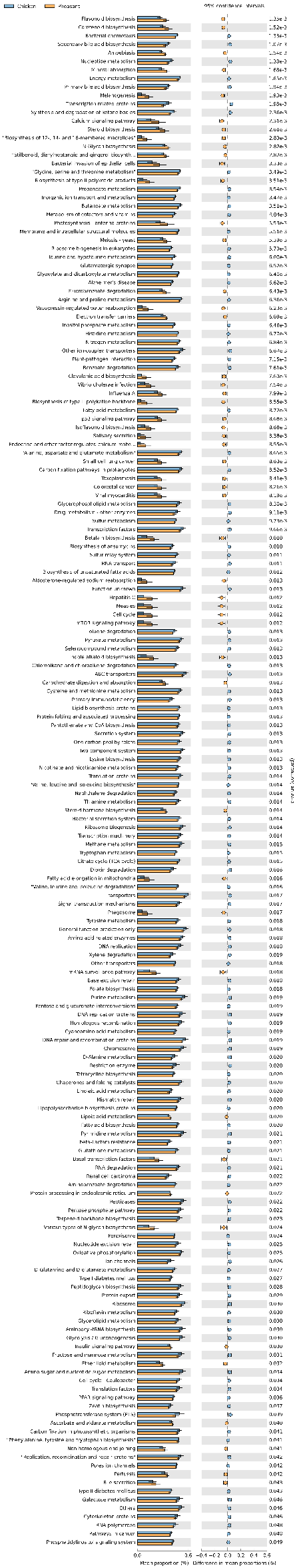


**Supplementary Figure 6** Prediction of microbial functions of the third levels between pheasants and chickens.

## Supplementary Tables

**Supplementary Table 1** Comparison of gut microbial genus abundance among the five pheasant lineages

|  | p value (* p <0.05, ** p < 0.01) | | | | | | | | | |
| --- | --- | --- | --- | --- | --- | --- | --- | --- | --- | --- |
| Genus | RN vs. MX | RN vs. PV | RN vs. SP | RN vs. MM | MX vs. PV | MX vs. SP | MX vs. MM | PV vs. SP | PV vs. MM | SP vs. MM |
| *Streptococcus* | 0.408 | 0.232 | 0.48 | 0.263 | 0.709 | 0.921 | 0.055 | 0.644 | 0.024* | 0.076 |
| *Halomonas* | 0.922 | 0.029* | 0.173 | 0.725 | 0.036* | 0.204 | 0.8 | 0.422 | 0.064 | 0.303 |
| *Ochrobactrum* | 0.019* | 0.465 | 0.655 | 0.4 | 0.098 | 0.007** | 0.121 | 0.249 | 0.911 | 0.209 |
| *Hymenobacter* | 0.033* | 0.018* | 0.014* | 0.015* | 0.797 | 0.675 | 0.736 | 0.866 | 0.936 | 0.928 |
| *Pseudomonas* | 0.681 | 0.224 | 0.563 | 0.334 | 0.106 | 0.329 | 0.576 | 0.54 | 0.032* | 0.132 |
| *Jonquetella* | 0.532 | 0.436 | 0.55 | 0.068 | 0.877 | 0.991 | 0.016* | 0.871 | 0.011* | 0.02* |
| *Bulleidia* | 0.723 | 0.665 | 0.969 | 0.093 | 0.937 | 0.701 | 0.044* | 0.645 | 0.037* | 0.11 |
| *Skermanella* | 0.039* | 0.029* | 0.024* | 0.024* | 0.897 | 0.792 | 0.835 | 0.891 | 0.938 | 0.951 |
| *Sphingomonas* | 0.221 | 0.901 | 0.378 | 0.879 | 0.271 | 0.042* | 0.283 | 0.317 | 0.978 | 0.304 |
| *Rhodococcus* | 0.903 | 0.027* | 0.695 | 0.807 | 0.036* | 0.61 | 0.714 | 0.012* | 0.015* | 0.877 |
| *Polaromonas* | 0.05* | 0.021* | 0.022* | 0.021* | 0.704 | 0.69 | 0.717 | 0.977 | 0.986 | 0.963 |
| *Rubellimicrobium* | 0.044* | 0.019* | 0.023* | 0.02* | 0.715 | 0.741 | 0.74 | 0.981 | 0.974 | 0.994 |
| *Limnohabitans* | 0.547 | 0.024* | 0.252 | 0.807 | 0.09 | 0.571 | 0.72 | 0.269 | 0.042* | 0.362 |
| *Devosia* | 0.972 | 0.043* | 0.598 | 0.925 | 0.04* | 0.575 | 0.952 | 0.141 | 0.035* | 0.536 |
| *Actinoplanes* | 0.014* | 0.007** | 0.009** | 0.008** | 0.801 | 0.796 | 0.809 | 0.989 | 0.992 | 0.982 |
| *Rubrivivax* | 0.133 | 0.082 | 0.039* | 0.045* | 0.804 | 0.526 | 0.592 | 0.694 | 0.773 | 0.91 |
| *Dyadobacter* | 0.011* | 0.028* | 0.028* | 0.033* | 0.695 | 0.746 | 0.638 | 0.955 | 0.937 | 0.893 |
| *Nocardioides* | 0.051 | 0.023* | 0.015* | 0.045* | 0.724 | 0.566 | 0.955 | 0.817 | 0.766 | 0.603 |
| *Deinococcus* | 0.128 | 0.136 | 0.041* | 0.068 | 0.975 | 0.554 | 0.75 | 0.534 | 0.726 | 0.778 |
| *Kineosporia* | 0.041* | 0.011* | 0.014* | 0.02* | 0.59 | 0.613 | 0.753 | 0.986 | 0.823 | 0.841 |
| *Amycolatopsis* | 0.511 | 0.298 | 0.088 | 0.267 | 0.093 | 0.021* | 0.081 | 0.477 | 0.945 | 0.519 |
| *Agrobacterium* | 0.056 | 0.221 | 0.078 | 0.021* | 0.473 | 0.914 | 0.664 | 0.554 | 0.251 | 0.596 |
| *Quadrisphaera* | 0.03* | 0.013* | 0.015* | 0.019* | 0.725 | 0.732 | 0.851 | 1 | 0.869 | 0.873 |
| *Chloronema* | 0.071 | 0.026* | 0.03* | 0.029* | 0.653 | 0.661 | 0.688 | 1 | 0.961 | 0.963 |
| *KSA1* | 0.04* | 0.021* | 0.021* | 0.021* | 0.785 | 0.731 | 0.785 | 0.938 | 1 | 0.938 |
| *Thiobacillus* | 0.468 | 0.207 | 0.312 | 0.439 | 0.05 | 0.757 | 0.961 | 0.028* | 0.045* | 0.794 |
| *Ralstonia* | 0.039* | 0.655 | 0.917 | 0.974 | 0.101 | 0.035* | 0.036* | 0.59 | 0.632 | 0.942 |
| *Sutterella* | 0.716 | 0.186 | 0.442 | 0.229 | 0.334 | 0.264 | 0.399 | 0.043* | 0.902 | 0.056 |
| *Novosphingobium* | 0.116 | 0.147 | 0.046* | 0.041* | 0.899 | 0.624 | 0.618 | 0.54 | 0.533 | 0.996 |
| *SAGMEG-1* | 0.041* | 0.02* | 0.016* | 0.032* | 0.756 | 0.656 | 0.909 | 0.886 | 0.844 | 0.738 |
| *Rathayibacter* | 0.099 | 0.045* | 0.041* | 0.041* | 0.706 | 0.642 | 0.677 | 0.921 | 0.968 | 0.952 |
| *Enhydrobacter* | 0.464 | 0.033* | 0.396 | 0.808 | 0.15 | 0.892 | 0.623 | 0.204 | 0.056 | 0.539 |
| *Roseococcus* | 0.013* | 0.002** | 0.002** | 0.002** | 0.53 | 0.481 | 0.488 | 0.924 | 0.947 | 0.976 |
| *Mycoplana* | 0.651 | 0.514 | 0.118 | 0.606 | 0.272 | 0.047* | 0.335 | 0.344 | 0.891 | 0.281 |
| *Fusobacterium* | 0.632 | 0.032* | 0.228 | 0.67 | 0.09 | 0.455 | 0.957 | 0.357 | 0.081 | 0.425 |
| *Truepera* | 0.042* | 0.061 | 0.028* | 0.035* | 0.867 | 0.809 | 0.933 | 0.686 | 0.801 | 0.873 |
| *Natronincola_Anaerovirgula* | 0.217 | 0.313 | 0.261 | 0.287 | 0.028* | 0.935 | 0.862 | 0.039* | 0.042* | 0.93 |
| *Phyllobacterium* | 0.126 | 0.539 | 0.491 | 0.393 | 0.353 | 0.032* | 0.491 | 0.202 | 0.808 | 0.132 |
| *Aerococcus* | 0.55 | 0.631 | 0.06 | 0.637 | 0.906 | 0.016* | 0.899 | 0.021* | 0.993 | 0.021* |
| *Jeotgalicoccus* | 0.387 | 0.631 | 0.055 | 0.715 | 0.698 | 0.007** | 614 | 0.019* | 0.907 | 0.024* |
| *Anaerobiospirillum* | 0.997 | 0.031* | 0.734 | 0.988 | 0.031* | 0.737 | 0.985 | 0.074 | 0.03* | 0.723 |
| *Rhodanobacter* | 0.945 | 0.022* | 0.694 | 0.533 | 0.026* | 0.645 | 0.579 | 0.01** | 0.089 | 0.319 |
| *Succinivibrio* | 0.601 | 0.039* | 0.873 | 0.635 | 0.115 | 0.727 | 0.962 | 0.062 | 0.105 | 0.763 |
| *Aequorivita* | 0.616 | 0.247 | 0.374 | 0.733 | 0.1 | 0.686 | 0.872 | 0.047* | 0.137 | 0.576 |
| *Planctomyces* | 0.788 | 0.036* | 0.502 | 0.746 | 0.065 | 0.352 | 0.554 | 0.008** | 0.017* | 0.721 |
| *Burkholderia* | 0.854 | 0.001** | 0.537 | 0.705 | 0.001** | 0.427 | 0.846 | 0.001** | 0.001** | 0.326 |
| *Modestobacter* | 0.027* | 0.017* | 0.021* | 0.024* | 0.848 | 0.875 | 0.956 | 0.976 | 0.891 | 0.917 |
| *Aminobacter* | 0.035* | 0.356 | 0.463 | 0.958 | 0.221 | 0.007** | 0.04* | 0.106 | 0.384 | 0.432 |
| *Curtobacterium* | 0.12 | 0.105 | 0.018* | 0.197 | 0.945 | 0.372 | 0.784 | 0.408 | 0.732 | 0.248 |
| *Sphingobium* | 0.962 | 0.026* | 0.857 | 0.813 | 0.029* | 0.893 | 0.776 | 0.046* | 0.015* | 0.681 |
| *Pseudonocardia* | 0.095 | 0.023* | 0.021* | 0.095 | 0.515 | 0.471 | 1 | 0.93 | 0.515 | 0.471 |
| *Knoellia* | 0.087 | 0.126 | 0.012* | 0.041* | 0.846 | 0.361 | 0.728 | 0.272 | 0.588 | 0.564 |
| *Rhizobium* | 0.035* | 0.497 | 0.432 | 0.868 | 0.142 | 0.006** | 0.023* | 0.151 | 0.398 | 0.532 |
| *Succinatimonas* | 0.683 | 0.067 | 0.528 | 0.794 | 0.027* | 0.814 | 0.505 | 0.018* | 0.113 | 0.377 |
| *Blastococcus* | 0.025* | 0.015* | 0.035* | 0.076 | 0.834 | 0.935 | 0.615 | 0.775 | 0.476 | 0.683 |
| *Kosmotoga* | 0.776 | 0.078 | 0.086 | 0.116 | 0.043* | 0.048* | 0.066 | 1 | 0.842 | 0.846 |
| *Sphingopyxis* | 0.29 | 0.241 | 0.619 | 0.66 | 0.029* | 0.591 | 0.532 | 0.104 | 0.11 | 0.944 |
| *Tissierella_Soehngenia* | 0.954 | 0.973 | 0.885 | 0.002** | 0.927 | 0.929 | 0.003** | 0.859 | 0.002** | 0.004** |
| *Dysgonomonas* | 0.802 | 0.173 | 0.194 | 0.484 | 0.263 | 0.289 | 0.344 | 0.976 | 0.042* | 0.051 |
| *Balneimonas* | 0.035* | 0.201 | 0.037* | 0.029* | 0.384 | 0.978 | 0.934 | 0.382 | 0.34 | 0.957 |
| *Pediococcus* | 0.702 | 0.601 | 0.109 | 0.571 | 0.887 | 0.05 | 0.854 | 0.037* | 0.966 | 0.034* |
| *Paracoccus* | 0.806 | 0.087 | 0.628 | 0.759 | 0.052 | 0.806 | 0.951 | 0.034* | 0.045* | 0.852 |
| *Roseburia* | 0.624 | 0.001** | 0.259 | 0.776 | 0.001** | 0.511 | 0.837 | 0.008** | 0.001** | 0.392 |
| *Rothia* | 0.527 | 0.295 | 0.781 | 0.021* | 0.675 | 0.734 | 0.085 | 0.456 | 0.188 | 0.046* |
| *Rhodobacter* | 0.676 | 0.075 | 0.782 | 0.912 | 0.167 | 0.495 | 0.598 | 0.046* | 0.059 | 0.865 |
| *Lysobacter* | 0.723 | 0.238 | 0.07 | 0.79 | 0.406 | 0.139 | 0.535 | 0.494 | 0.15 | 0.04* |
| *Lachnospira* | 0.802 | 0.017* | 0.76 | 0.554 | 0.032* | 0.951 | 0.733 | 0.042* | 0.068 | 0.786 |
| *Peptostreptococcus* | 0.711 | 0.893 | 0.022* | 0.813 | 0.613 | 0.009** | 0.893 | 0.03* | 0.711 | 0.013* |
| *Ensifer* | 0.683 | 0.035* | 0.637 | 0.384 | 0.013* | 0.94 | 0.641 | 0.013* | 0.004** | 0.705 |
| *Rheinheimera* | 0.463 | 0.047* | 0.769 | 0.713 | 0.197 | 0.315 | 0.273 | 0.027* | 0.02* | 0.949 |
| *Dechloromonas* | 0.758 | 0.062 | 0.834 | 0.823 | 0.115 | 0.611 | 0.933 | 0.044* | 0.098 | 0.669 |
| *Nesterenkonia* | 0.751 | 0.751 | 0.016* | 0.821 | 1 | 0.034* | 0.928 | 0.034* | 0.928 | 0.027* |
| *Luteimonas* | 0.273 | 0.004** | 0.856 | 0.618 | 0.06 | 0.374 | 0.546 | 0.008** | 0.015* | 0.761 |
| *Coriobacterium* | 0.694 | 0.844 | 0.865 | 0.017* | 0.844 | 0.831 | 0.043* | 0.983 | 0.028* | 0.03* |
| *Prochlorococcus* | 0.58 | 0.046* | 0.251 | 0.656 | 0.142 | 0.538 | 0.913 | 0.409 | 0.116 | 0.471 |

**Supplementary Table 2** Comparison of gut microbial species abundance in the five pheasant lineages.

|  | p value (* p <0.05, ** p < 0.01) | | | | | | | | | |
| --- | --- | --- | --- | --- | --- | --- | --- | --- | --- | --- |
| Species | RN vs. MX | RN vs. PV | RN vs. SP | RN vs. MM | MX vs. PV | MX vs. SP | MX vs. MM | PV vs. SP | PV vs. MM | SP vs. MM |
| *s__adhaesivum* | 0.098 | 0.003** | 0.001** | 0.098 | 0.206 | 0.093 | 0.88 | 0.588 | 0.11 | 0.031* |
| *s__alactolyticus* | 0.131 | 0.005** | 0.458 | 0.809 | 0.153 | 0.331 | 0.216 | 0.007** | 0.012* | 0.65 |
| *s__alcaligenes* | 0.532 | 0.086 | 0.03* | 0.219 | 0.203 | 0.235 | 0.746 | 0.523 | 0.223 | 0.146 |
| *s__anthropi* | 0.213 | 0.333 | 0.807 | 0.299 | 0.759 | 0.297 | 0.022* | 0.451 | 0.042* | 0.179 |
| *s__atrovinosus* | 0.515 | 0.085 | 0.035* | 0.052 | 0.139 | 0.029* | 0.072 | 0.321 | 0.657 | 0.639 |
| *s__aureofaciens* | 0.818 | 0.021* | 0.584 | 0.137 | 0.077 | 0.564 | 0.283 | 0.017* | 0.501 | 0.097 |
| *s__aureum* | 0.402 | 0.365 | 0.11 | 0.868 | 0.086 | 0.02* | 0.62 | 0.436 | 0.379 | 0.157 |
| *s__biforme* | 0.125 | 0.027* | 0.032* | 0.74 | 0.201 | 0.353 | 0.143 | 0.568 | 0.03* | 0.028* |
| *s__brevis* | 0.217 | 0.011* | 0.88 | 0.005** | 0.103 | 0.224 | 0.08 | 0.018* | 0.821 | 0.011* |
| *s__bryophila* | 0.185 | 0.016* | 0.797 | 0.365 | 0.119 | 0.329 | 0.698 | 0.036* | 0.075 | 0.554 |
| *s__cereus* | 0.788 | 0.17 | 0.487 | 0.122 | 0.131 | 0.628 | 0.266 | 0.118 | 0.006** | 0.817 |
| *s__columnare* | 0.96 | 0.601 | 0.054 | 0.096 | 0.723 | 0.198 | 0.215 | 0.029* | 0.046* | 0.999 |
| *s__debontii* | 0.183 | 0.841 | 0.751 | 0.328 | 0.149 | 0.08 | 0.023* | 0.932 | 0.469 | 0.469 |
| *s__difficile* | 0.133 | 0.87 | 0.516 | 0.368 | 0.028* | 0.29 | 0.618 | 0.244 | 0.201 | 0.702 |
| *s__dispar* | 0.176 | 0.003** | 0.011* | 0.147 | 0.018* | 0.066 | 0.788 | 0.64 | 0.038* | 0.121 |
| *s__distasonis* | 0.08 | 0.169 | 0.04* | 0.928 | 0.78 | 0.64 | 0.155 | 0.96 | 0.202 | 0.093 |
| *s__firmus* | 0.034* | 0.668 | 0.519 | 0.722 | 0.058 | 0.006** | 0.009** | 0.262 | 0.399 | 0.736 |
| *s__genomosp.* | 0.404 | 0.601 | 0.951 | 0.046* | 0.667 | 0.358 | 0.003** | 0.537 | 0.004** | 0.046* |
| *s__genosp.* | 0.156 | 0.751 | 0.667 | 0.765 | 0.059 | 0.018* | 0.047* | 0.935 | 0.969 | 0.892 |
| *s__granulorum* | 0.187 | 0.004** | 0.007** | 0.059 | 0.011* | 0.02* | 0.406 | 0.531 | 0.055 | 0.095 |
| *s__halophobica* | 0.089 | 0.029* | 0.031* | 0.268 | 0.755 | 0.812 | 0.465 | 0.893 | 0.237 | 0.245 |
| *s__hathewayi* | 0.28 | 0.755 | 0.557 | 0.081 | 0.45 | 0.131 | 0.648 | 0.398 | 0.18 | 0.033* |
| *s__kestanbolensis* | 0.689 | 0.083 | 0.017* | 0.929 | 0.038* | 0.006** | 0.781 | 0.598 | 0.086 | 0.022* |
| *s__komagatae* | 0.548 | 0.416 | 0.069 | 0.95 | 0.194 | 0.046* | 0.607 | 0.115 | 0.425 | 0.097 |
| *s__mrcj* | 0.933 | 0.012* | 0.021* | 0.032* | 0.018* | 0.033* | 0.04* | 0.531 | 0.911 | 0.785 |
| *s__muciniphila* | 0.04* | 0.018* | 0.67 | 0.048* | 0.343 | 0.01** | 0.924 | 0.009** | 0.405 | 0.016* |
| *s__mucosae* | 0.595 | 0.046* | 0.645 | 0.147 | 0.019* | 0.179 | 0.042* | 0.06 | 0.304 | 0.165 |
| *s__multivorum* | 0.054 | 0.045* | 0.979 | 0.002** | 0.801 | 0.072 | 0.158 | 0.061 | 0.313 | 0.003** |
| *s__nasimurium* | 0.256 | 0.344 | 0.093 | 0.047* | 0.966 | 0.709 | 0.302 | 0.81 | 0.4 | 0.426 |
| *s__plantarum* | 0.412 | 0.033* | 0.014* | 0.045* | 0.054 | 0.007** | 0.086 | 0.691 | 0.883 | 0.584 |
| *s__producta* | 0.036* | 0.854 | 0.861 | 0.438 | 0.027* | 0.012* | 0.127 | 0.585 | 0.46 | 0.242 |
| *s__pseudoalcaligenes* | 0.339 | 0.095 | 0.007** | 0.285 | 0.308 | 0.073 | 0.961 | 0.765 | 0.309 | 0.063 |
| *s__roseus* | 0.78 | 0.879 | 0.31 | 0.112 | 0.615 | 0.456 | 0.166 | 0.077 | 0.019* | 0.184 |
| *s__stercoris* | 0.307 | 0.439 | 0.438 | 0.19 | 0.904 | 0.053 | 0.025* | 0.128 | 0.054 | 0.5 |
| *s__subflava* | 0.18 | 0.864 | 0.071 | 0.179 | 0.261 | 0.854 | 0.023* | 0.197 | 0.344 | 0.011* |
| *s__triangulatus* | 0.025* | 0.058 | 0.234 | 0.081 | 0.259 | 0.121 | 0.219 | 0.359 | 0.828 | 0.485 |
| *s__viridiflava* | 0.461 | 0.359 | 0.092 | 0.513 | 0.166 | 0.348 | 0.748 | 0.027* | 0.085 | 0.162 |
| *s__yabuuchiae* | 0.163 | 0.957 | 0.92 | 0.001** | 0.302 | 0.442 | 0.024* | 0.905 | 0.007** | 0.025* |

**Supplementary Table3** Comparison of gut microbial genus abundance between pheasants and chickens.

| taxonomy | Relative fold change | p value | taxonomy | Relative fold change | p value |
| --- | --- | --- | --- | --- | --- |
| *Streptococcus* | 5.68 | 3.31E-15 | *rc4-4* | 1.24 | 0.001 |
| *ph2* | 4.67 | 1.26E-19 | *Sporosarcina* | 1.24 | 0.001 |
| *Peptoniphilus* | 4.35 | 1.56E-19 | *Aerococcus* | 1.23 | 0.003 |
| *1-68* | 3.83 | 2.72E-17 | *Rhodoplanes* | 1.22 | 8.70E-04 |
| *Bacillus* | 3.62 | 5.66E-25 | *Achromobacter* | 1.21 | 7.24E-04 |
| *Devosia* | 3.41 | 9.75E-20 | *Rubellimicrobium* | 1.18 | 0.021 |
| *5-7N15* | 3.40 | 1.64E-14 | *Lachnospira* | 1.16 | 0.019 |
| *Megamonas* | 3.11 | 1.63E-06 | *Meiothermus* | 1.15 | 0.010 |
| *Megasphaera* | 3.08 | 3.70E-16 | *Carnobacterium* | 1.14 | 0.017 |
| *Mycoplasma* | 2.50 | 1.80E-12 | *Curtobacterium* | 1.13 | 0.005 |
| *CF231* | 2.50 | 7.67E-13 | *Paracoccus* | 1.11 | 0.023 |
| *Collinsella* | 2.21 | 1.18E-06 | *Blastococcus* | -1.11 | 0.011 |
| *Avibacterium* | 2.17 | 2.83E-09 | *Planomicrobium* | -1.11 | 0.005 |
| *Dialister* | 2.13 | 4.57E-11 | *Cloacibacterium* | -1.12 | 0.009 |
| *Helcococcus* | 2.07 | 1.48E-08 | *Ensifer* | -1.13 | 0.014 |
| *Methylibium* | 2.05 | 7.92E-20 | *Kribbella* | -1.13 | 0.005 |
| *Bulleidia* | 2.05 | 2.18E-05 | *Acetobacter* | -1.13 | 0.004 |
| *Ralstonia* | 1.93 | 4.85E-13 | *Brevibacillus* | -1.13 | 0.005 |
| *Enterococcus* | 1.89 | 2.70E-04 | *Craurococcus* | -1.13 | 0.006 |
| *Sulcia* | 1.88 | 2.03E-10 | *Clostridium* | -1.14 | 0.006 |
| *Deinococcus* | 1.85 | 2.48E-13 | *Balneimonas* | -1.14 | 4.86E-04 |
| *Pseudomonas* | 1.81 | 9.21E-05 | *Flavihumibacter* | -1.15 | 0.004 |
| *Vibrio* | 1.79 | 1.41E-09 | *Schlegelella* | -1.16 | 0.001 |
| *Glaciecola* | 1.77 | 7.58E-10 | *Modestobacter* | -1.16 | 3.31E-04 |
| *Hymenobacter* | 1.71 | 9.07E-08 | *Hydrogenophilus* | -1.16 | 9.28E-05 |
| *Phascolarctobacterium* | 1.67 | 7.01E-12 | *Acidovorax* | -1.17 | 3.13E-05 |
| *Pseudoramibacter_Eubacterium* | 1.30 | 0.007 | *Candidatus Rhabdochlamydia* | -1.18 | 1.39E-06 |
| *Brevundimonas* | 1.61 | 1.37E-10 | *Curvibacter* | -1.20 | 1.20E-06 |
| *Rubrivivax* | 1.61 | 1.21E-10 | *Holdemania* | -1.24 | 0.007 |
| *LCP-6* | 1.61 | 3.23E-06 | *Xanthomonas* | -1.34 | 2.46E-11 |
| *Chryseobacterium* | 1.60 | 2.69E-11 | *Butyricimonas* | -1.39 | 0.013 |
| *Agrobacterium* | 1.57 | 1.93E-09 | *Sulfurimonas* | -1.40 | 0.010 |
| *Photobacterium* | 1.57 | 9.21E-08 | *Anaerofustis* | -1.45 | 4.45E-05 |
| *Roseburia* | 1.51 | 5.20E-07 | *Flavisolibacter* | -1.50 | 0.012 |
| *Anaerobiospirillum* | 1.51 | 7.56E-04 | *Bifidobacterium* | -1.57 | 0.008 |
| *Dyadobacter* | 1.51 | 5.62E-08 | *Methanobrevibacter* | -1.59 | 0.004 |
| *DA101* | 1.49 | 1.35E-07 | *Dehalobacterium* | -1.62 | 1.88E-04 |
| *Shewanella* | 1.46 | 3.23E-07 | *Phenylobacterium* | -1.66 | 0.015 |
| *Nevskia* | 1.46 | 8.91E-06 | *Helicobacter* | -1.68 | 4.07E-04 |
| *Leuconostoc* | 1.45 | 7.78E-06 | *YRC22* | -1.71 | 0.008 |
| *Succinivibrio* | 1.45 | 4.45E-05 | *Oscillospira* | -1.75 | 5.52E-04 |
| *Skermanella* | 1.44 | 1.76E-05 | *Parabacteroides* | -1.77 | 0.015 |
| *Methylobacterium* | 1.42 | 0.001 | *cc_115* | -1.83 | 0.013 |
| *Polaromonas* | 1.41 | 4.64E-06 | *Zoogloea* | -1.86 | 7.69E-05 |
| *Erwinia* | 1.41 | 0.011 | *Methanocorpusculum* | -1.86 | 3.96E-04 |
| *Rhodanobacter* | 1.41 | 2.99E-06 | *Campylobacter* | -1.87 | 0.007 |
| *Varibaculum* | 1.39 | 1.89E-04 | *Mucispirillum* | -1.88 | 0.002 |
| *Mycobacterium* | 1.39 | 6.28E-07 | *Wautersiella* | -1.97 | 1.83E-04 |
| *Sphingobium* | 1.38 | 4.20E-06 | *Methyloversatilis* | -2.12 | 0.001 |
| *Candidatus Portiera* | 1.33 | 7.72E-04 | *Candidatus Azobacteroides* | -2.32 | 1.82E-04 |
| *Pseudoalteromonas* | 1.32 | 2.35E-04 | *Bacteroides* | -2.34 | 1.86E-05 |
| *Proteus* | 1.31 | 2.84E-04 | *Blvii28* | -2.37 | 0.002 |
| *GW-34* | 1.63 | 5.85E-05 | *Candidatus Arthromitus* | -2.51 | 8.52E-04 |
| *Nocardioides* | 1.30 | 5.02E-04 | *Dechloromonas* | -2.71 | 5.10E-04 |
| *Anaerococcus* | 1.30 | 0.007 | *Turicibacter* | -2.92 | 8.89E-05 |
| *Desulfococcus* | 1.29 | 5.38E-04 | *Vestibaculum* | -2.97 | 3.55E-07 |
| *Sphingobacterium* | 1.29 | 2.66E-04 | *Barnesiella* | -2.99 | 1.16E-05 |
| *Micrococcus* | 1.28 | 6.42E-05 | *Jonquetella* | -3.02 | 0.006 |
| *Tissierella_Soehngenia* | 1.27 | 0.006 | *Odoribacter* | -3.13 | 3.97E-05 |
| *Pelobacter* | 1.27 | 0.002 | *Sneathia* | -3.18 | 1.62E-04 |
| *Planctomyces* | 1.25 | 0.001 | *AF12* | -3.32 | 1.35E-04 |
| *Aeriscardovia* | -8.91 | 8.86E-06 | *Lactobacillus* | -4.18 | 0.003 |
| *Porphyromonas* | -16.66 | 4.57E-06 | NOTE:+Pheasant>Chicken,- Pheasant<Chicken | | |

**Supplementary Table4** Summary of sequencing data for all samples.

| Sample | Effective sequences | High quality sequences | The ratio of high quality sequence and effective sequence |
| --- | --- | --- | --- |
| RN1 | 80,973 | 76,317 | 94.25% |
| RN2 | 45,734 | 43,928 | 96.05% |
| RN3 | 44,282 | 43,492 | 98.22% |
| RN4 | 111,153 | 103,929 | 93.50% |
| RN5 | 70,793 | 65,733 | 92.85% |
| RN6 | 70,824 | 66,462 | 93.84% |
| RN7 | 89,491 | 82,397 | 92.07% |
| RN8 | 90,750 | 82,667 | 91.09% |
| RN9 | 65,868 | 64,799 | 98.38% |
| RN10 | 105,988 | 100,620 | 94.94% |
| MX1 | 183,156 | 180,463 | 98.53% |
| MX2 | 88,614 | 83,478 | 94.20% |
| MX3 | 55,466 | 51,393 | 92.66% |
| MX4 | 103,921 | 96,915 | 93.26% |
| MX5 | 64,950 | 59,659 | 91.85% |
| MX6 | 82,593 | 78,199 | 94.68% |
| MX7 | 85,655 | 81,164 | 94.76% |
| MX8 | 97,176 | 88,272 | 90.84% |
| MX9 | 91,470 | 79,604 | 87.03% |
| MX10 | 57,005 | 55,966 | 98.18% |
| PV1 | 27,303 | 27,006 | 98.91% |
| PV2 | 102,856 | 90,275 | 87.77% |
| PV3 | 114,346 | 111,838 | 97.81% |
| PV4 | 94,472 | 93,549 | 99.02% |
| PV5 | 57,863 | 51,319 | 88.69% |
| PV6 | 164,176 | 157,165 | 95.73% |
| PV7 | 104,066 | 102,288 | 98.29% |
| PV8 | 102,997 | 101,275 | 98.33% |
| PV9 | 78,077 | 61,966 | 79.37% |
| PV10 | 52,925 | 48,044 | 90.78% |
| SP1 | 187,611 | 178,569 | 95.18% |
| SP2 | 106,933 | 104,826 | 98.03% |
| SP3 | 48,221 | 45,571 | 94.50% |
| SP4 | 72,549 | 63,784 | 87.92% |
| SP5 | 29,294 | 28,815 | 98.36% |
| SP6 | 36,884 | 36,164 | 98.05% |
| SP7 | 148,046 | 144,763 | 97.78% |
| SP8 | 48,619 | 46,862 | 96.39% |
| SP9 | 34,521 | 31,684 | 91.78% |
| MM1 | 90,156 | 85,815 | 95.19% |
| MM2 | 128,495 | 119,133 | 92.71% |
| MM3 | 96,504 | 94,343 | 97.76% |
| MM4 | 103,527 | 98,626 | 95.27% |
| MM5 | 91,602 | 86,214 | 94.12% |
| MM6 | 107,394 | 100,022 | 93.14% |
| MM7 | 87,223 | 85,910 | 98.49% |
| MM8 | 99,071 | 96,740 | 97.65% |
| MM9 | 76,172 | 74,670 | 98.03% |
| MM10 | 57,761 | 56,450 | 97.73% |
| Total | 4,235,526 | 4,009,143 | 94.57% |
